# Clinical implications of the interaction between PD-1/PD-L1 and PI3K/AKT/mTOR pathway in progression and treatment of non-small cell lung cancer

**DOI:** 10.7150/jca.77619

**Published:** 2022-10-03

**Authors:** Zihan Quan, Yang Yang, Hongmei Zheng, Yuting Zhan, Jiadi Luo, Yue Ning, Songqing Fan

**Affiliations:** Department of Pathology, The Second Xiangya Hospital, Central South University, Changsha, Hunan, 410011, China.

**Keywords:** PD-1/PD-L1, PI3K/AKT/mTOR pathway, Inhibitors, immunotherapy

## Abstract

The discovery of immune checkpoints has been well known to provide novel clues for cancer treatments. Immunotherapy against the programmed cell death protein-1 (PD-1) /programmed death-ligand-1 (PD-L1), one of the most popular auxiliary treatments in recent years, has been applied in various tumor treatments, including non-small cell lung cancer (NSCLC). However, inevitable issues such as side effects and drug resistance emerge following the use of immune checkpoint inhibitors. The PI3K/AKT/mTOR pathway may participate in the regulation of PD-L1 expression. Abnormal PI3K/AKT/mTOR pathway activation results in increased PD-L1 protein translation, whereas PD-L1 overexpression can activate the PI3K/AKT/mTOR pathway inversely. Via downstream proteins, including 4E-BP1, STAT3, NF-κB, c-MYC, and AMPK in aberrant energy status, the PI3K/AKT/mTOR pathway can regulate PD-L1 post-transcription and translation. Besides, the regulation of the PI3K pathway by the PD-1/PD-L1 axis involves both tumor cells and the tumor immune microenvironment. Inhibitors targeting the PD-1/PD-L1 have been successfully applied in the treatment of gastrointestinal cancer and breast cancer. Meanwhile, drug resistance from alternative pathway activation also evidently affects clinical progress. To achieve a better therapeutic effect and quality of survival, the combination of multiple treatment modalities presents great research value. Here we reviewed the interaction between PD-1/PD-L1 and PI3K/AKT/mTOR pathway in the progression and treatment of NSCLC and summarized its clinical implications. The intracellular interactions between PD-1/PD-L1 and the PI3K/AKT/mTOR pathway indicate that PD-1/PD-L1 inhibitors have a wide range of potential applications. And we presented the mechanism for combining therapy with monoclonal antibody PD-1/PD-L1 and PI3K/AKT/mTOR inhibitors in this review, to broaden the therapies for NSCLC.

## Introduction

Lung cancer, primary non-small cell lung cancer (NSCLC), has ranked second in morbidity and first in mortality among cancer diseases worldwide, constructing a serious threat to human life and health 1. With further study of the mechanism of tumorigenesis and the development of molecular detection, programmed cell death protein-1 (PD-1) /programmed death-ligand-1 (PD-L1) monoclonal antibodies have become the first-line therapy for advanced NSCLC patients 2. Furthermore, the investigation of immunotherapy has become popular in cancer therapeutic research. Although some patients with PD-L1^+^ NSCLC have benefited from the application of PD-1/PD-L1 monoclonal antibodies markedly 3, 4, the side effects and drug resistance were still of great concern to patients 4. Therefore, for patients with PD-L1^+^ in the advanced stage of NSCLC, combination therapy has become a considerable option, such as the combination of PD-1 monoclonal antibody pembrolizumab with platinum agents' chemotherapy 5. The clinical trials of the third-generation EGFR-TKI osimertinib combined with PD-L1 monoclonal antibody durvalumab have achieved good experimental results 6. The PI3K/AKT/mTOR pathway is an essential intracellular signaling pathway that regulates the processes of cancer diseases including cell metabolism, cell proliferation, apoptosis, and gene expression 7. The PI3K/AKT/mTOR pathway is also reported to participate in the immunosurveillance of the tumor microenvironment 8. Inhibitors of the PI3K/AKT/mTOR pathway have been under development and in clinical trials 9. In this review, we will discuss the clinical implications of the correlation between PD-1/PD-L1 and PI3K/AKT/mTOR pathway activation in the progression and treatment of NSCLC, and explore the current situation of relevant targeted drugs and immunotherapy for NSCLC.

## PD-L1 expression and PD-1 activation on tumor immunosuppression

NSCLC tumor cells usually express PD-L1 on the membrane and its receptor PD-1 is expressed on the membrane of CD8^+^ T cells 10. The coexpression of PD-L1 and PD-1 inactivates CD8^+^ T cells 11, thereby suppressing anti-tumor immune activity 12. Researchers find that the activation of PD-1 by PD-L1 inhibits transduction via inactivating the co-receptor CD28 13. In addition, CD4^+^Foxp3+ regulatory T cells (Tregs) belong to the immunosuppressive subpopulation of CD4^+^ T cells 14, while PD-1 expressed on their surface maintains the immunosuppressive function and enhances immune tolerance. CD4^+^ T cells are induced to differentiate towards Tregs by the activation of PD-1. And the high expression of Foxp3 mainly through inhibition of mTOR (mammalian target of rapamycin) increases the immunosuppressive effect as well 15. It has been proved that several mechanisms encoded by CD274 can regulate the expression of PD-L1 16, intracellular factors, and tumor microenvironment 17. For example, PTEN influences PD-L1 expression by regulating the mRNA levels, while NF-κB induces PD-L1 gene transcription by directly binding to its promoter and other indirect ways 18. Plenty of factors and pathways have been found to play a critical role in immunotherapy; more details are still under experiment exploration.

## Anti-PD-1/PD-L1 immunotherapy for NSCLC patients with advanced stage

**Figure 1:**
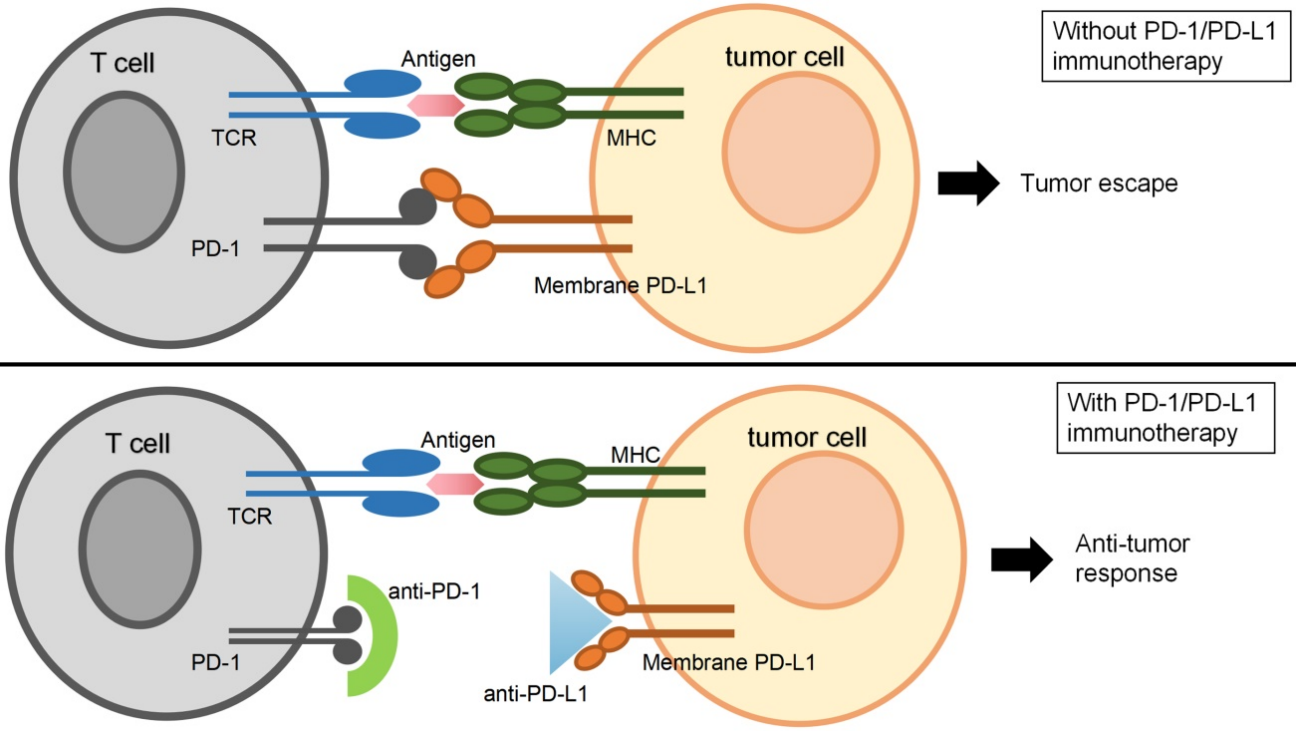
** The schematic of PD-1/PD-L1 immunotherapy.** T lymphocyte (T cell) plays an important role in killing cancer cells when surface T cell receptors (TCR) recognize and bind to the major histocompatibility complex (MHC) molecules. Programmed death ligand 1 (PD-L1, CD274) is a kind of immune checkpoint protein, which is highly expressed in part of tumor cells and promotes tumor cell escape from being killed by T-cell. Programmed death 1 (PD-1) expressed on T-lymphocytes can bind to PD-L1 and inhibits T cell proliferation and activity. When anti-PD-L1 drugs antagonized PD-L1 or anti-PD-1 drugs antagonized PD-1, the anti-tumor immune function of T cells recovered.

The anti-tumor immune function of T cells can be regained via anti-PD-1/PD-L1 drugs (Figure 1). For NSCLC patients with positive PD-L1 expression, immune checkpoint inhibitor therapy improves the patient's overall survival (OS) rate compared to traditional chemotherapy 19. Immune checkpoint inhibitors now in clinical use or trials mainly contain the PD-1 monoclonal antibody, such as nivolumab and pembrolizumab 20, and the PD-L1 monoclonal antibody, including atezolizumab and durvalumab 21, 22. Clinical trial studies have found that the median remission period and median OS significantly increase by using all those four drugs, and compared to conventional chemotherapy, the grade 3/4 adverse events (AEs) are significantly shortened to varying degrees 3, 4.

However, the application of a single PD-1 or PD-L1 monoclonal antibody remains flawed. First of all, the indications for only using one type of monoclonal antibody are limited 2, 23. Nivolumab has been recommended in the NCCS guidelines as a follow-up treatment for metastatic non-squamous NSCLC after first-line chemotherapy or the tumor made progression after chemotherapy 2. FDA has approved pembrolizumab as first-line therapy for patients with metastatic NSCLC and with PD-L1 expression levels ≥50% (with EGFR mutations, negative or unknown ALK rearrangement test results also available) 24. Meanwhile, no contraindications (e.g., severe autoimmune disease or organ transplantation) are also required in these patients 25. In one follow-up treatment for NSCLC 26, different from pembrolizumab, the use of atezolizumab required disease-related information about the patient, rather than the PD-L1 expression level detection. Phase III clinical trial of durvalumab has reported that those NSCLC patients who are unable to apply tumor resection surgically and with PD-L1 ≥25% using durvalumab after radiotherapy obtain significantly better prognosis than those receiving radiotherapy alone 27. Secondly, tumor cells present resistance to immune checkpoint inhibitors 28 with natural and acquired resistance to PD-1 and PD-L1 inhibitors in various tumors such as melanoma, NSCLC, kidney cancer, and so on 29. In addition, a certain degree of drug toxicity and side effects exist in any kind of PD-1 or PD-L1 monoclonal antibodies 30. Therefore, as a popular research topic in recent years 31, the combination therapy of medicine shows great research value and clinical significance for broadening the scope of indications for immune checkpoint inhibitors, alleviating drug resistance, and mitigating the side effects of anti-cancer drugs including targeted drugs 32. At this stage, the combination of immune checkpoint inhibition therapy with targeted drugs such as EGFR-TKIs 33 or platinum-agent chemotherapy is a popular way of combining therapies for intermediate to advanced NSCLC 34.

## Roles of PI3K/AKT/mTOR pathway in tumor development

PI3K/AKT/mTOR pathway upstream gene PIK3CA amplification and PI3K, AKT mutations have been found in NSCLC tissues. The expression of all of these genes increased, while PTEN gene expression is absent, compared to paracancerous tissues 35. Activation of the PI3K/AKT/mTOR pathway, which is related to multiple upstream and downstream elements, is associated with oncogenesis.

PI3K is an intracellular signal transduction protein with phosphatidylinositol 3- kinase activity, thus Class I PI3Ks are closely associated with cancer, and the PIK3CA gene is involved in encoding the subunits associated with this protein 36. Overexpression of the PIK3CA gene can directly hyperactivate the PI3K/AKT/mTOR pathway 37. The activation of PI3K is associated with overexpressed EGFR caused by EGFR gene mutations. The ERBB3 protein from the EGFR receptor tyrosine kinase family also drives PI3K activation. In some cases, EGFR family-related proteins in EGFR-mutant NSCLC can activate PI3K through roles of GAB adaptor proteins, independent of ERBB3 protein 38. PI3K phosphorylates PIP2 and generates PIP3 then further activates AKT (protein kinase B) 39. The activation of AKT phosphorylates corresponding enzymes and kinases and regulates a variety of downstream signaling pathways 40, which will indirectly promote the expression of the mammalian target of rapamycin protein (mTOR) 41. On the contrary, PTEN promotes the conversion of PIP3 to PIP2 42. Moreover, the silence of the PTEN gene blocks the conversion of PIP3 to PIP2 43 and enhances AKT activation 44. MTOR is an element in two different multiprotein signaling complexes, mTORC1, and mTORC2, both involved in mediating apoptosis and proliferation in different ways 44. Because of the complexity of the PI3K/AKT/mTOR pathway in regulating cell proliferation and apoptosis-related responses, inhibition of each of the responses in this pathway tends to activate the paracrine pathway, leading to the development of drug resistance 45.

## Interaction between PI3K/AKT/mTOR pathway and PD-1/PD-L1

**Figure 2:**
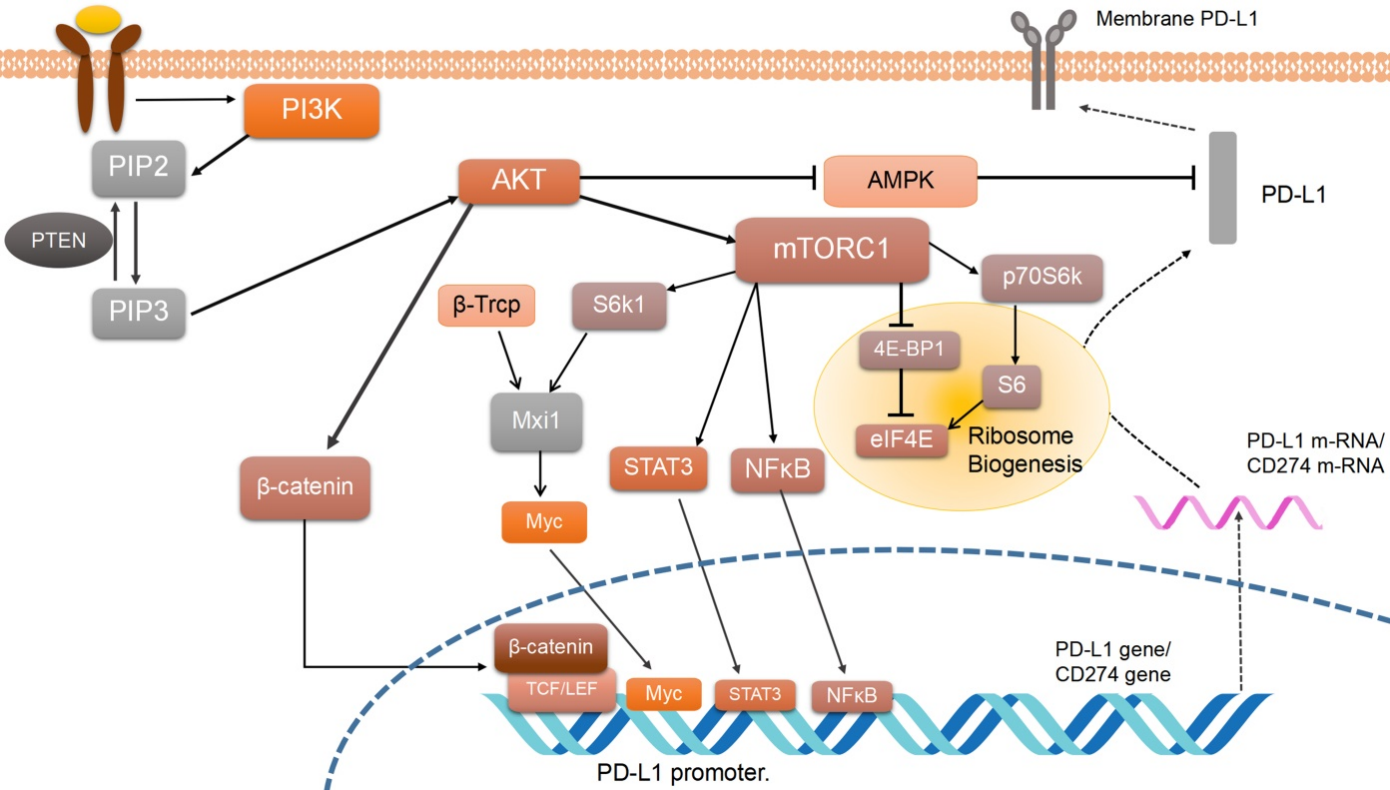
** PI3K/AKT/mTOR pathway regulating PD-L1 expression.** PD-L1 expression is regulated by the PI3K/AKT/mTOR pathway, which controls numerous cell processes including PD-L1 translation and post-transcription. Arrows mean activation, and bars mean inhibition. While dotted lines represent PD-L1 expression and solid lines represent PI3K pathway functions. Phosphatidylinositol 3-kinases (PI3K) is activated by various factors including growth factor receptor tyrosine kinases. PI3K promotes phosphatidylinositol 4, 5-bisphosphate (PIP2) to generate phosphatidylinositol 3, 4, 5-trisphosphate (PIP3), and then activates Protein Kinase B (AKT). AKT mediated PD-L1 regulation is divided into two parts, activating β-catenin and activating the mechanistic target of rapamycin complex 1(mTORC1). β-catenin enhances PD-L1 expression by activating T-cell factor/lymphoid enhancing factor (TCF/LEF) and combining with the PD-L1 promoter. mTORC1 regulates PD-L1 promoter through signal transducer and activator of transcription 3(STAT3), mammalian target of rapamycin (NF-κB), and Myc, activated by beta-transducin repeat-containing protein (β-TrCP) and ribosomal protein S6 kinase beta-1(S6K1) mediated by MAX interactor 1(Mxi1) degradation. Also, mTOC1 induces ribosome biogenesis by promoting p70 protein S6 kinase beta-1(p70S6K1) and inhibiting eukaryotic translation initiation factor 4E-binding protein 1(4E-BP1). Both ways upregulate eukaryotic translation initiation factor 4E (elF4E), which promotes PD-L1 mRNA translation.

The PI3K/AKT/mTOR pathway can control PD-L1 expression. In lung squamous carcinoma or lung adenocarcinoma tissues with mutations in NRAS, KRAS, EGFR, BARF, PIK3CA, EML4-ALK, activation of PI3K/AKT/mTOR pathway and PD-L1 expression can be detected simultaneously 46. PI3K/AKT/mTOR-related inhibitors (e.g. mTOR inhibitor rapamycin) decreased PD-L1 expression 47, while stimulation of enhanced AKT/mTOR expression increases PD-L1 expression (validated in mouse experiments) 48. Multiple responses are linked between PD-L1 and PI3K/AKT/mTOR (Figure 2). For example, in lung squamous carcinoma, deletion of the PTEN gene is found to lead to higher PD-L1 protein translation, while PTEN gene expression deficiency simultaneously promoted AKT activation 49. As downstream elements of AKT, β-catenin/TCF/LEF transcription complex stimulates CD274 gene transcription by binding to the PD-L1 promoter 50. Meanwhile, the AKT downstream signaling protein NF-κB also acts on its promoter to induce PD-L1 mRNA expression 18.

On the other hand, molecules like transcription factors including c-Myc 51 and transcription activating factor STAT3 may play a role in the post-transcription of PD-L1 52^.^ STAT3 phosphorylation is relevant to mTOR, but it remains unclear whether mTOR suppressed or promoted STAT3 activity. Some researchers reveal that the phosphorylation of STAT3 by mTOR leads to its maximal activation 53. As for c-Myc, it has been reported that Mxi1/S6k/β-Trcp can activate c-Myc by promoting Mxi1 degradation and then work on downstream factors such as the CD274 gene 54. One of the most important mechanisms of mTORC1 is regulating its downstream molecular S6 kinase. High expression of p70 S6 kinase also plays an important role in controlling the expression of PD-L1. The overexpression of mTORC1 can negatively regulate PD-L1 expression, while it suppresses β-TrCP-mediated proteasomal degradation of PD-L1 55. Besides, the activation of p70S6K can also promote the translation efficiency of PD-L1 mRNA in the ribosome via activating 4E-BP1. So at least four ways have been mentioned above to regulate PD-L1 expression. One of them is involved in regulating ribosome biogenesis and translation efficiency 56, while another one is referred to as the Mxi1/S6k/β-Trcp pathway 53. It is supposed that the mTOR pathway may regulate PD-L1 expression through many of the ways mentioned above.

**Figure 3:**
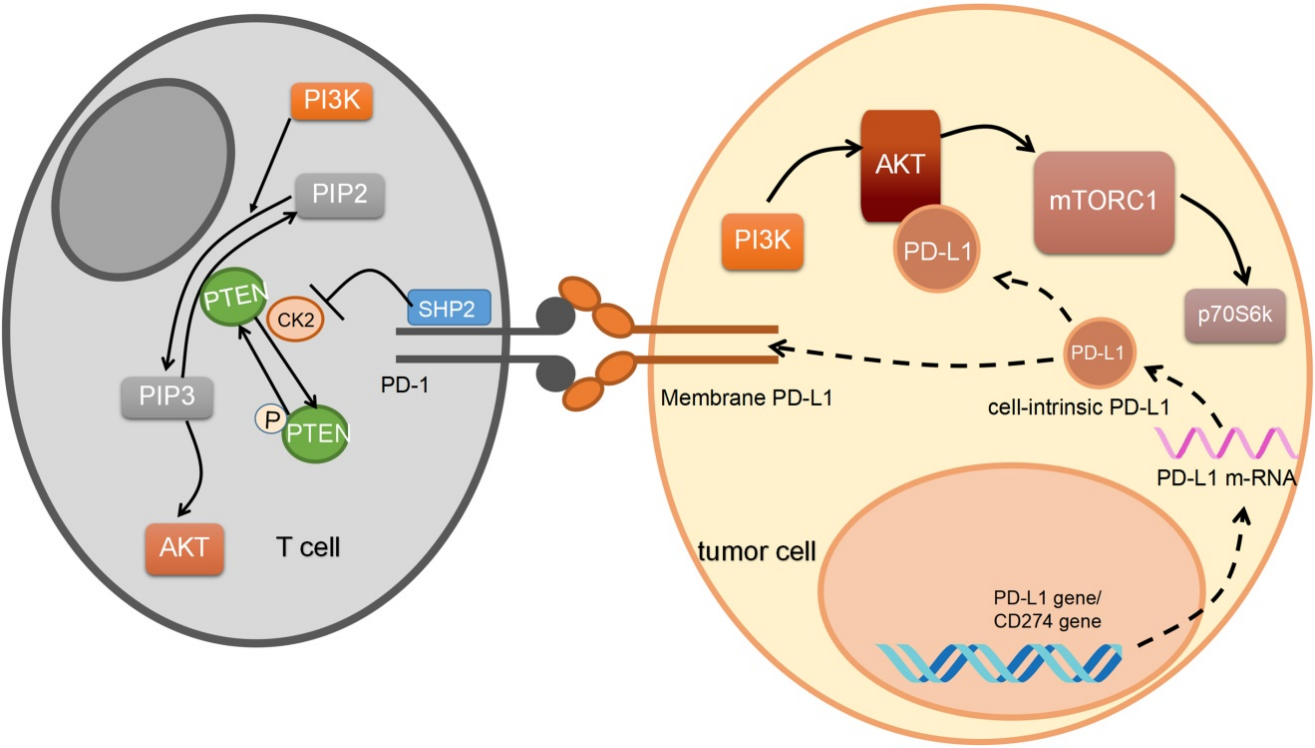
** PD-1/PD-L1 regulates PI3K pathway.** The PD-1/PD-L1 regulates a number of functions of PI3K pathway. Both the transmembrane part and intracellular part of the PD-1/PD-L1 pathway participate in this process. Reactions can happen in both immune cells and tumor cells. Arrows mean activation, and bars mean inhibition. While dotted lines represent PD-L1 expression. In tumor cells, after PD-L1 mRNA translation, cell-intrinsic PD-L1 activates Protein Kinase B (AKT). After PD-1/PD-L1 binding, Src homology-2-containing protein tyrosine phosphatase 2(SHP2) promotes Phosphatase and tensin homolog (PTEN) phosphorylation through working on casein kinase 2(CK2), further promoting phosphatidylinositol 3, 4, 5-trisphosphate (PIP3) to generate phosphatidylinositol 4, 5-bisphosphate (PIP2).

In turn, PD-1/PD-L1 can also regulate the PI3K/AKT/mTOR pathway (Figure 3). PD-L1 activates the PI3K/AKT pathway by stabilizing β-catenin 57, and overexpression of PD-L1 also increases the expression of p-AKT. For example, in gastric cancer, researchers confirm that the PD-1/PD-L1 axis can upregulate AKT phosphorylation 58. According to another study, in glioma, cell-intrinsic PD-L1 binds to AKT preferentially when compared to other PI3K/AKT signal proteins. Suppressing cell-intrinsic PD-L1 then decreases phosphorylation of mTORC1 and p70S6K in melanoma 59 and ovarian cancer cells 60.

By the way, AMP-activated protein kinase (AMPK) has been reported to be involved in regulating the interaction between PI3K/AKT/mTOR pathway 61 and PD-1/PD-L1 because of aberrant energy status in cancer. Energy deprivation can affect anti-tumor immunity, induce AMPK to phosphorylate PD-L1, and decrease PD-L1 protein abundance 62. On one hand, activated AMPK phosphorylates PD-L1 on its Ser283 site to block the combination of PD-L1 with CMTM4, a positive regulator of PD-L1, to induce its degradation 62. On the other hand, researchers demonstrate that the activated AMPK phosphorylated S195 site of PD-L1 results in its abnormal glycosylation and degeneration 63. Also, a previous study verifies that activated AKT can phosphorylate AMPK directly on its Ser485 site or indirectly on its Thr172 site 64, and down-regulate its expression. In conclusion, activating PI3K/AKT pathway can suppress the AMPK function and then induces a higher PD-L1 expression.

PD-1/PD-L1 activates the PI3K/AKT pathway not just in tumor cells but also in the immune microenvironment. It has been found in breast cancer that PD-1 on an element termed myeloid-driven suppressor cells (MDSC) immune microenvironment bound to PD-L1 on B cell can activate PI3K/AKT/NF-κB signaling pathway in B cell 65. Then B cell stimulation hindered T cell immune response and promoted tumor cells' immune escape. On the contrary, in T cells, PD-1 collected downstream molecular SHP-2 which suppresses PI3K activation through targeting PTEN phosphorylation mediated by CK2 66. The phosphorylated PTEN is in a stable situation, resulting in lower PI3K/AKT expression. Although there is no direct evidence that high expression of PD-L1 is simply mTOR-dependent, it is well known that AKT/mTOR pathway activation can promote the immune escape of cancer cells by promoting high PD-L1 expression.

## Value-added regulation of tumor cells by PI3K/AKT/mTOR pathway inhibitors

Several PI3K/AKT/mTOR pathway targeted therapeutic drugs mainly target related genes such as PIK3CA, AKT, TSC1/2, mTOR, PTEN, and so on 67. Since the development of pathway targeted drugs lags behind the tumorigenesis mechanism research, currently many of them are still in clinical trials (Table 1). At this stage, drugs targeting PI3K are inhibitors of various PI3K isoforms. For example, alpelisib, a drug targeting PIK3CA mutant breast cancer in phase Ⅱ clinical trials 68 displays antitumor activity in pre-initiation studies. Copanlisib and duvelisib are FDA-approved for marketing for specific types of lymphoma or leukemia in the United States. ACP-319 (acertapharma), BYL719 (novartis), and serabelisib are also in clinical trials, but cytotoxic response occurs evidently with each of those drugs used alone 69. Another discovery indicates that the antipsychotic agent flupentixol can inhibit lung cancer development via inducing apoptosis of oncocytes 70.

AKT inhibitors can be divided into two types: AKT competitive inhibitors and AKT aliasing inhibitors. The former competitively inhibits the ATP binding site on AKT and prevents AKT activation. While the latter inhibits AKT activation and phosphorylation by altering the chemical structure of the AKT PH structural domain, thereby preventing AKT localization on the cell membrane. AKT competitive inhibitors are majorly listed below: Capivasertib (AZD5363) 71, a selective PAN-AKT inhibitor that entered clinical trials for the treatment of breast, gastric, and prostate cancers; Afuresertib (GSK2110183) 72, a monotherapy of relapsed or refractory multiple myeloma treatment; Uprosertib (GSK2141795), which remains in phase Ⅰ and Ⅱ studies 73; and the AKT inhibitor Ipatasertib (GDC-0068, RG7440), a monotherapy for the treatment of triple-negative breast cancer, still being in phase Ⅰ and Ⅱ studies 74. Perifosine as an inhibitor of AKT metaplasia to inhibit neuroblastoma tumor cell growth has entered phase Ⅱ studies 75. Meanwhile, the development of AKT inhibitors still encounters plenty of predicaments. The most notable one is that AKT plays a significant role in maintaining the dynamic balance of cellular physiological functions in normal tissues. AKT inhibitors can cause an unavoidable cytotoxic effort on normal tissues in cancer patients 76. In addition, like other targeted drugs, the single use of AKT inhibitor tends to induce drug resistance and the substitution related to tumor formation.

There are three generations of mTOR inhibitors 77. The first generation of mTOR inhibitors includes rapamycin, also known as sirolimus, approved by the FDA as an immunosuppressant and primarily used to prevent immune rejection in organ transplantation 78. Ridaforolimus also belongs to the first-generation mTOR inhibitors 79. Everolimus, and temsirolimus have little effect on mTORC2 80. Single-agent application of mTOR inhibitor such as everolimus has been applied to treat advanced neuroendocrine tumors, breast cancer, and non-functioning gastrointestinal, and temsirolimus is used to treat lymphangioleiomyomatosis 81. The second-generation mTOR inhibitors such as Torin1 82, work on ATP binding sites to block kinase activity of both TORC1 and TORC2 proteins, and PI-103, targets ATP binding sites of mTOR and PI3K. Whereas, drug development is still in the clinical research stage 83. In contrast, newly developed third-generation mTOR inhibitors named rapalink-1 show potential usage in patients with first- and second-generation drugs resistant tumors 84.

Similar to other target agents, resistance can also happen when using PI3K/AKT/mTOR pathway targeted drugs 85. After using mTOR inhibitors, for example, resistance occurs because of the activation of other tumor-related pathway elements and downstream of mTOR. An experiment shows that after being treated with everolimus or AZD8055, the mice obtained markedly increased activation in EGFR and MEK-ERK signaling pathway in tumor epithelial and stromal cells, respectively 86. In the PI3K/AKT/mTOR pathway downstream, suppressing the expression or activation of mTOR may lead to the decrease of 4E-BP1 expression. 4E-BP1 suppresses eIF4E expression, and the overexpression of 4E-BP1 will make tumor cells sensitive to rapamycin as eIF4E plays a critical role in controlling translation 87 and tumor progression 88. In summary, the new generation of target drug development and new combination therapies are under exploration 89.

## Anti-tumor immune effects of PI3K/AKT/mTOR pathway inhibitors

The effect of the PI3K/AKT/mTOR pathway on immune cells and the immune microenvironment is complicated and combined with multiple pathways. PI3K/AKT/mTOR inhibitors affect the PD-L1 expression in cancer cells 8. In NSCLC, the application of rapamycin results in a decrease in PD-L1 expression 47. In the presence of interferon-γ (IFN-γ), inhibition of PI3K enhances the antitumor effect of IFN-γ, while IFN-γ expression positively is correlated with tumor infiltration of CD3^+^ T cells. However, IFN-γ also activates AKT/mTOR pathway in cancer diseases, and induces PD-L1 expression, antagonizing its antitumor effect 90. In consideration of the cytotoxicity and resistance when single-use, PI3K/AKT/mTOR pathway inhibitors are of research interest in combination with other targeted drugs, such as the combination of inhibitors targeting two different components of the pathway. For example, the clinical trials of everolimus combined with EGFR-TKI for the treatment of advanced NSCLC show no significant improvement in therapeutic efficacy compared to EGFR-TKI alone. Nevertheless, the application of everolimus is still suggested for patients with EGFR-TKI-resistant NSCLC 91. Therefore, further clinical trials of PI3K/AKT/mTOR pathway inhibitors in combination with targeted agents are needed.

PI3K/AKT/mTOR inhibitors also influence the antitumor effects of tumor immune cells infiltrating in cancers 92. In the tumor environment, PI3Kγ protein expression inhibits NF-κB activity through AKT and mTOR while stimulating C/EBPβ activation in macrophages, resulting in suppression of antitumor immune effects 93. Selective inhibition of macrophage PI3Kγ stimulates CD8^+^ T cell activation and enhances cytotoxic effects. Activating PI3K-mTOR signaling in T cells in the tumor environment suppresses autoimmunity by inhibiting activation and differentiation of common T cells and specializing in CD4+Foxp3+ regulatory T cells (Tregs) 94. PI3K/AKT/mTOR inhibitors restore the anti-tumor immune effect of the body to some extent by blocking pathway activation.

## PI3K/AKT/mTOR pathway and anti-tumor immunotherapy

As mentioned above, PI3K/AKT/mTOR pathway activation is closely related to PD-L1 expression and impacts the tumor immune microenvironment 95^.^ The application of PD-L1 monoclonal antibodies enhances the antitumor immune effects of macrophages by inhibiting the AKT-mTOR pathway 96. PD-L1 inhibitors have antagonistic effects on AKT and ERK1/2 activation to inhibit tumor proliferation 97. So that blocking PD-L1 with antibodies in gastrointestinal mesenchymal tumors (GIST) can reduce CD8^+^ T cell depletion by regulating the PI3K/AKT/mTOR pathway to play an antitumor immune role 98. Researchers find that in triple-negative breast cancer, atezolizumab can inhibit the mTOR signaling pathway by affecting P53-related genes 99^.^ At the same time, both PD-1/PD-L1 monoclonal antibodies and PI3K/AKT/mTOR pathway inhibitors may develop resistance through activation of the bypass pathway, and have drug toxicity and side effects when achieving significant cancer suppression 100. Overall, the combination drug application is a therapeutic modality that will be investigated further (Table 2). For instance, the combination of rapamycin and anti-PD-1 antibody has dampened the progression of NSCLC 47, with the pharmacological effect of rapamycin on inhibiting the activation of the AKT/mTOR pathway from differentiating CD3^+^ T cells 101. While both drugs can alleviate the increased production of regulatory T cells (Tregs), PD-L1 enhances the role of Everolimus in the treatment of renal cell carcinoma 102.

## Conclusion

This review discussed the tumor immunosuppressive effect of PD-1/PD-L1 inhibitors and the fundamental scenario of immune checkpoint inhibition therapy with the application of monoclonal antibodies. Taking NSCLC as an example, the review explained the components of the PI3K/AKT/mTOR signaling pathway and described their functions in driving carcinogenesis and suppressing antitumor immunity respectively. We also introduced the relevant immunosuppressive agents including the role and situation of single agent use in anti-tumor represented by everolimus, as well as the feasibility of combining multiple targeted agents and multiple adverse medication effects. The viability of a therapeutic strategy combining PI3K/AKT/mTOR pathway inhibitors with PD-1/PD-L1 inhibition will be considered. To date, research into PI3K/AKT/mTOR signaling pathway inhibitors is still currently in progress, and it exhibits great positive significance to investigate the interaction between PD-L1 expression and PI3K/AKT/mTOR signaling pathway activation for addressing anticancer drug resistance, prolonging tumor patient survival, and improving patient prognosis.

## Figures and Tables

**Table 1:** Inhibitors of the PI3K/AKT/mTOR pathway in clinical development

Inhibitors	Target(s)	Tumor	Study phase	References
Alpelisib	PI3Kα	Advanced Solid Tumor	II	NCT01387321
Duvelisib	PI3K	lymphoma or leukemia	II	NCT04707079
Novartis (BYL719 )	PI3Kα	SCC of the head and neck	II	NCT01602315
		breast cancer	II	NCT02506556
Serabelisib (INK1117, TAK117, MLN1117)	PI3Kα	Metastatic Solid Tumors	I	NCT01449370
Flupentixol	ATP binding area of PI3Kα	lung cancer	Preclinical	70
Capivasertib (AZD5363)	AKT	B-NHL	II	NCT05008055
Afuresertib (GSK2110183)	AKT	Solid tumors, Hematologic malignancies	II	NCT01531894
Uprosertib (GSK2141795)	AKT	Solid Tumors, Lymphoma	I	NCT01266954
Ipatasertib (GDC-0068, RG7440)	AKT	Solid tumor	I	NCT04341259
Perifosine	AKT	Neuroblastoma tumor, RCC, NSCLC	II	NCT00399789, NCT00399789
Everolimus (RAD001)	mTORC1	advanced NET, breast cancer, MM, non-functioning GI, pulmonary NENs	II (lung, MM)	NCT00401778, NCT00770120
Temsirolimus	mTORC1	RCC, LAM, lung cancer	II (lung, HL)	NCT00093782, NCT00838955
Torin1	ATP binding site of mTORC1 and mTORC2	not mentioned	Preclinical	86
PI-103	ATP binding sites of mTOR and PI3K	AML, glioblastoma, melanoma	Preclinical	87

**Abbreviations:** NCT, ClinicalTrials.gov. No.; SCC, squamous cell carcinoma; B-NHL, B-cell Non-Hodgkin lymphoma; NSCLC, non-small cell lung cancer; GI, gastrointestinal; NENs, pulmonary neuroendocrine neoplasms; MM, malignant mesothelioma; NET, neuroendocrine tumor; LAM, lymphangioleiomyomatosis; AML, acute myeloid leukemia; RCC, renal cell carcinoma; HL, Hodgkin's lymphoma.

**Table 2:** Combination therapy of PI3K/AKT/mTOR inhibitors with PD-1/PD-L1 monoclonal antibody

PI3K/AKT/mTOR Inhibitors	Target	PD-1/PD-L1 monoclonal antibody	Tumor	Study phase	References
ABI-009	mTOR	Nivolumab	mTOR Activating Mutated ES, PEComa, DT, Chordoma, NSCLC, UC, Melanoma, RCC, SCC, HCC, cHL, CRC	I and II	NCT03190174
Sirolimus	mTOR	Durvalumab	NSCLC	I	NCT04348292
Ipatasertib	AKT	Atezolizumab	Metastatic or Locally Advanced Malignancies	II	NCT04551521
Copanlisib	PI3K	Nivolumab	Unresectable or MSS Solid Tumor, MSS Colon Cancer	I and II	NCT03711058
Duvelisib (VS-0145, Copiktra)	PI3K	Pembrolizumab (Keytruda)	R/M HNSCC	I and II	NCT04193293
SF1126	PI3K	Nivolumab	AHCC	I	NCT03059147

**Abbreviations:** ES, Ewing Sarcoma; PEComa, perivascular epithelioid cell tumor; NSCLC, non-small cell lung cancer; DT, Desmoid Tumor; UC, urothelial carcinoma; RCC, renal cell carcinoma; SCC, squamous cell carcinoma; HCC, Hepatocellular Carcinoma; cHL, Classical Hodgkin Lymphoma; CRC, Colorectal Cancer; MSS, Microsatellite Stable; R/M, recurrent or metastatic; HNSCC, head and neck squamous cell carcinoma; AHCC, Advanced Hepatocellular Carcinoma.
